# Supplementary material for: Optimizing Thermoelectric Performance of Hybrid Crystals Bi2O2Se1−xTex in the Bi2O2X System
Source: Materials (Basel). 2024 Mar 26;17(7):1509. doi: 10.3390/ma17071509 (PMC11012295; doi:10.3390/ma17071509)
Supplement: Supplementary file 1 [file materials-17-01509-s001.zip › materials-2934743-supplementary.pdf]

Supporting information

Optimizing Thermoelectric Performance of Mixed Crystals $Bi_2O_2Se_{1-x}Te_x$ in the Bi_2O_2Se/Bi_2O_2Te Pseudobinary Bulk System

Fan Xie, Zhiyao Ma, Jian Zhou

School of Materials Science and Engineering, Key Laboratory for Polymeric Composite and Functional Materials of Ministry of Education, Guangzhou Key Laboratory of Flexible Electronic Materials and Wearable Devices, Laboratory of Advanced Electronics and Fiber Materials, Sun Yat-sen University, Guangzhou, Guangdong, 510275 China

Email: zhouj296@mail.sysu.edu.cn (Jian Zhou)

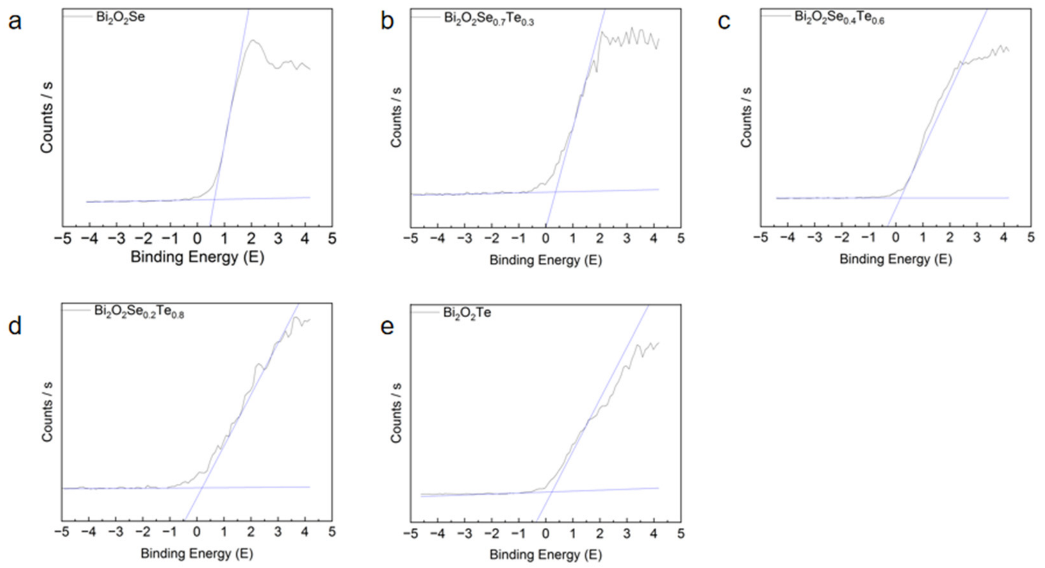

Figure S1. The valence band of samples. The valence band of Bi_2O_2Se(a), $Bi_2O_2Se_{0.7}Te_{0.3}$(b), $Bi_2O_2Se_{0.4}Te_{0.6}$(c), $Bi_2O_2Se_{0.2}Te_{0.8}$(d) and Bi_2O_2Te(e).

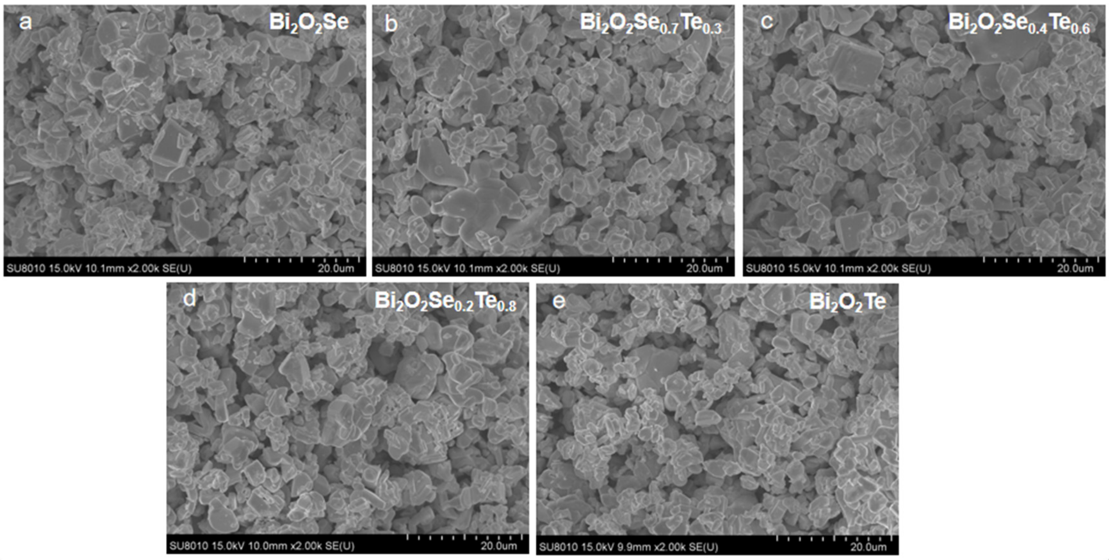

Figure S2. SEM of samples. SEM images of all $Bi_2O_2Se_{1-x}Te_x$ samples including Bi_2O_2Se (a), $Bi_2O_2Se_{0.7}Te_{0.3}$ (b), $Bi_2O_2Se_{0.4}Te_{0.6}$ (c), $Bi_2O_2Se_{0.2}Te_{0.8}$ (d) and Bi_2O_2Te (e). We measured the average size of each sample from the SEM plots, and the average size of these particles was calculated to be 3.4±0.7, 3.2±0.9, 3.9±1.0, 4.2±0.8, and 2.6±0.6 μm for different Te doping levels of $Bi_2O_2Se_{1-x}Te_x$ (x=0, 0.2, 0.4, 0.7, 1).

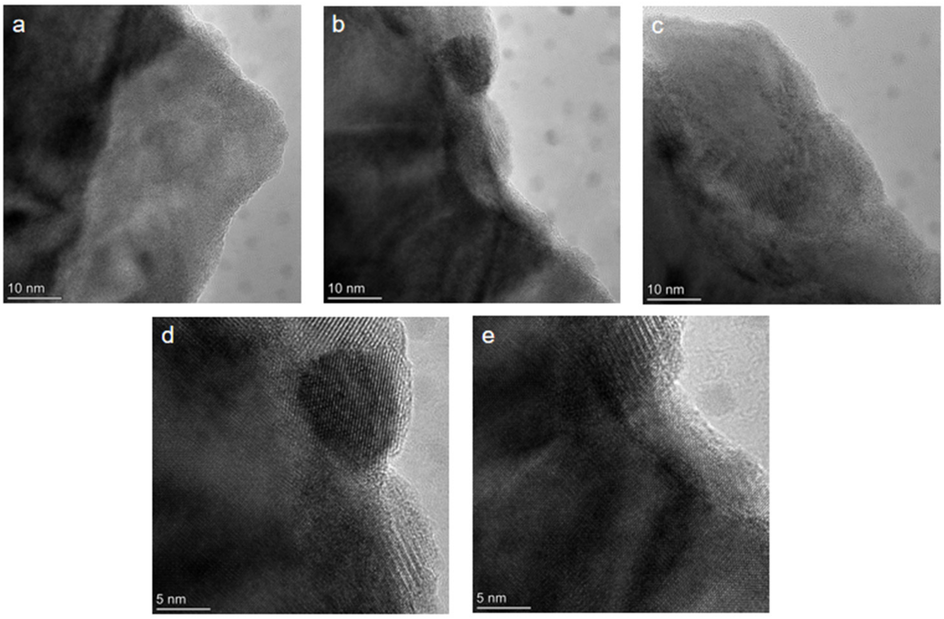

Figure S3. (**a-e**) TEM of $Bi_2O_2Se_{0.4}Te_{0.6}$. TEM images of $Bi_2O_2Se_{0.4}Te_{0.6}$ compounds.

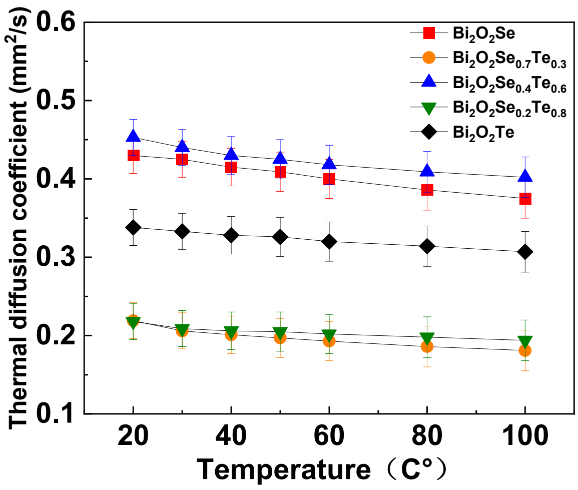

Figure S4. The thermal diffusion coefficient of Bi_2O_2Se, $Bi_2O_2Se_{0.7}Te_{0.3}$, $Bi_2O_2Se_{0.4}Te_{0.6}$, $Bi_2O_2Se_{0.2}Te_{0.8}$ and Bi2O2Te at different temperatures.

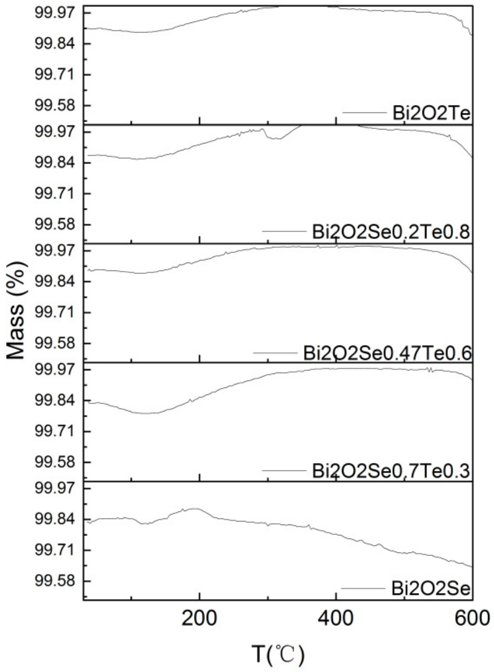

Figure S5. The TGA show the relationship between Mass and temperature of Bi_2O_2Se, $Bi_2O_2Se_{0.7}Te_{0.3}$, $Bi_2O_2Se_{0.4}Te_{0.6}$, $Bi_2O_2Se_{0.2}Te_{0.8}$ and Bi2O2Te. It means that the material is able to efficiently convert waste heat into electricity in a variety of practical scenarios, while maintaining long-term stability and reliability.

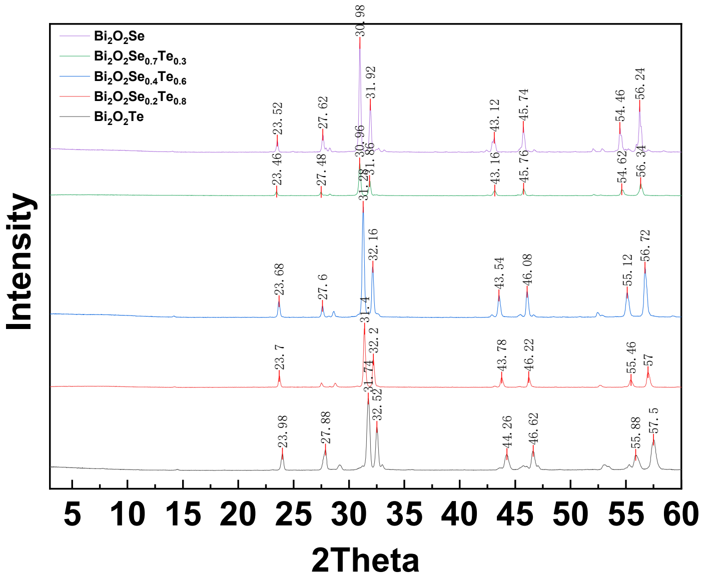

Figure S6. XRD of samples Bi_2O_2Se, $Bi_2O_2Se_{0.7}Te_{0.3}$, $Bi_2O_2Se_{0.4}Te_{0.6}$, $Bi_2O_2Se_{0.2}Te_{0.8}$ and Bi_2O2Te after heating at 600°C.

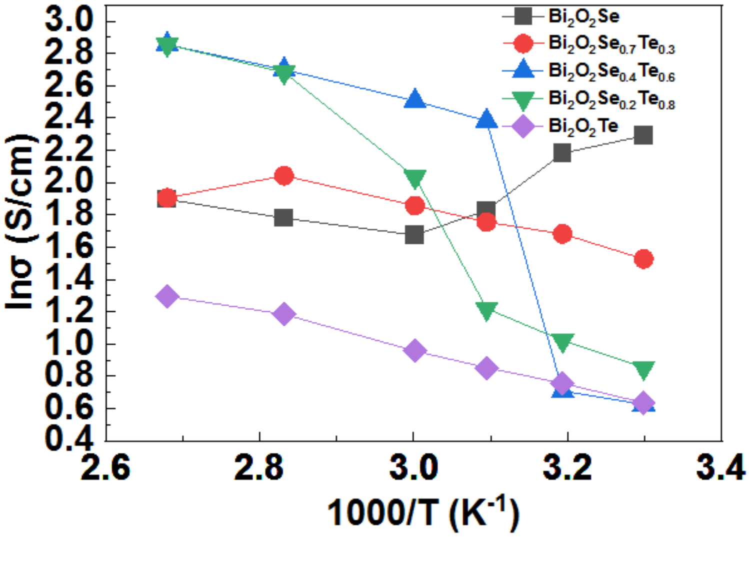

Figure S7. Arrhenius plot of samples.
